# Does morphological structure modulate access to embedded word meaning in child readers?

**DOI:** 10.3758/s13421-021-01164-3

**Published:** 2021-03-22

**Authors:** Jana Hasenäcker, Olga Solaja, Davide Crepaldi

**Affiliations:** International School for Advanced Studies (SISSA), via Bonomea 265, 34136 Trieste, Italy

**Keywords:** Visual word recognition, Reading development, Morphological processing, Embedded word identification, Semantic categorization

## Abstract

Beginning readers have been shown to be sensitive to the meaning of embedded neighbors (e.g., CROW in CROWN). Moreover, developing readers are sensitive to the morphological structure of words (TEACH-ER). However, the interaction between orthographic and morphological processes in meaning activation during reading is not well established. What determines semantic access to orthographically embedded words? What is the role of suffixes in this process? And how does this change throughout development? To address these questions, we asked 80 Italian elementary school children (third, fourth, and fifth grade) to make category decisions on words (e.g., is CARROT a type of food?). Critically, some target words for no-answers (e.g., is CORNER a type of food?) contained category-congruent embedded stems (i.e., CORN). To gauge the role of morphology in this process, half of the embedded stems were accompanied by a pseudosuffix (CORN-ER) and half by a non-morphological ending (PEA-CE). Results revealed that words were harder to reject as members of a category when the embedded stem was category-congruent. This effect held both with and without a pseudosuffix, but was larger for pseudosuffixed words in the error rates. These results suggest that orthographic stems are activated and activation is fed forward to the semantic level regardless of morphological structure, followed by a decision-making process that might strategically use suffix-like endings.

## Introduction

Learning to activate meaning from abstract symbols is one of the core achievements of reading acquisition. How exactly this skill develops in children is still not well understood (cf. Nation, 2009). Previous studies have shown that beginning readers already access semantic information from orthographically embedded neighbors, like CROW in CROWN (Nation & Cocksey, 2009). At the same time, developing readers become sensitive to the morphological structure of words (e.g., DEAL–ER; e.g., Burani et al., 2002). Orthographic and morphological processes seem to interact during visual word identification (e.g., Grainger & Ziegler, 2011). In the present study, we address the use of orthographic and morphological information in word-meaning activation in children.

The role of morphology in child reading has gained increased attention in recent years. Many studies investigated the role of morphological awareness, that is, the ability to manipulate morphemes, in reading acquisition (for a review, see Kuo & Anderson, 2006). Such studies have shown that elementary school children are aware of the morphological structure of words and can use this knowledge to learn new complex words (e.g., Bertram et al., 2000), spell complex words correctly (e.g., Deacon & Bryant, 2006), and determine their meaning (e.g., Krott & Nicoladis, 2005).

Furthermore, a number of studies have used lexical decision and naming tasks to examine the role of morphological structure in word reading during the elementary school years in several languages (e.g., Angelelli et al., 2014; Burani et al., 2002; Carlisle & Stone, 2005; Casalis et al., 2015; Dawson et al., 2018; Hasenäcker et al., 2017). In these tasks, children are not asked to show awareness of the existence of meaningful word parts or to manipulate them in any way. More simply, their behavior is compared across different types of morphologically simple and complex words. For example, Burani et al. (2008) showed that Italian children were faster and more accurate in reading suffixed words (CASS-IERE, cash-ier) as compared to matched simple words (CAMMELLO, camel). The same was true for morphological pseudowords (DONN-ISTA, woman-ist) compared to simple pseudowords (DENNOSTO). These findings indicate that morpheme-based reading is especially useful for developing readers. Moreover, Traficante et al. (2011) compared the role of stems and suffixes in reading aloud and found that stems, rather than suffixes, provided a head-start to the decomposition of new words. To investigate the activation of stems in suffixed words (e.g., TEACH in TEACHER), the masked priming paradigm (Forster & Davis, 1984) has also been used. Interestingly, this paradigm has allowed researchers to also focus on words like CORNER or IRONY, which have the surface appearance of morphological complexity (CORN+ER, IRON+Y), but whose meaning has nothing to do with their pseudostems (CORN, IRON). These stimuli go under the name of *pseudosuffixed* words in the literature and are especially interesting because they exhibit a mismatch between the meanings of the orthographic subunit and the meaning of the whole word unit. They have been widely investigated in masked priming studies with adults (e.g., Feldman et al., 2015; Longtin et al., 2003; Rastle et al., 2004; Rastle & Davis, 2008), but only in a few studies with children (but see Beyersmann et al., 2012; Schiff et al., 2012; Quémart & Casalis, 2015; Quémart et al., 2011) and only in one study were they presented overtly, that is, not as masked primes (Amenta et al., 2015). There is general agreement that children activate embedded stems in the presence of real suffixes (e.g., TEACH in TEACHER), but it is less clear whether they also activate embedded stems in the presence of pseudosuffixes (e.g., CORN in CORNER) and in words where the final letter chunk never works as a suffix in the language (*nonsuffixes*; e.g., EW in CASH–EW). A closely related body of research has used suffixed and nonsuffixed nonwords, like SPORTIFY and SPORTINT, in masked priming to investigate children’s sensitivity to embedded stems in the presence and absence of suffixes (Beyersmann et al., 2015; Hasenäcker et al., 2016; Hasenäcker, Beyersmann, & Schroeder, 2020a). These studies have yielded additional evidence that beginning readers already identify embedded stems in the absence of morphological structure.

Based on the findings discussed above, Grainger and Beyersmann (2017) proposed a theoretical framework of *embedded stem activation*, which includes a developmental perspective. According to this account, activating embedded stems in written words serves as a bootstrapping mechanism to acquire a morphological decomposition system that takes into account the overall morphological structure of words (see also Beyersmann et al., 2019). Only once this morphological decomposition system is in place later in development does the principle of “full decomposition” (i.e., the fact that a word can be exhaustively decomposed into morphemes, like CORN+ER) lead to differences in the automatic parsing of pseudosuffixed and nonsuffixed words, such as CORNER and CASHEW. Hence, according to the embedded stem account, morphological (pseudo)structure does not affect the automatic activation of orthographically embedded stems in young readers’ word recognition at a prelexical level (Grainger & Beyersman, 2017). However, this does not preclude morphological structure being considered at later stages of processing. In fact, the embedded stem account assumes that morphological structure plays a crucial role as a result of feedback from morpho-semantic representations. This combination of morphology-blind embedded stem activation and morphologically structured feedback serves as a tool for children to process unfamiliar words containing a known embedded word, understand their meanings, and thus expand their vocabulary through reading.

Importantly, most of the studies above focused on processing at the lexico-orthographic level (how quickly children identify an existing word form) or on phonology (how quickly children read a word aloud), whereas the semantic level (how children access the meaning of a word) has been comparatively neglected. However, *understanding* is the ultimate goal of reading. Hence, specifying the role of morphology from orthographic all the way up to semantic activation is of major importance (Nation, 2009). From the perspective of the embedded stem account, one could expect that the more semantics is required from the task, the more morphological structure should modulate embedded word activation.

Semantic categorization tasks are an appropriate way to elicit semantic access of a stimulus word. In a key study, Nation and Cocksey (2009) used a semantic categorization task to show that beginning readers already activate meaning from embedded words. Participants were 7-year-old English-speaking children. The authors used the paradigm of Bowers, Davis, and Hanley (2005), in which participants had to make decisions on the category membership of target words (i.e., is CROWN a bird?). Importantly, some no-answers had orthographically embedded words that were actually congruent with the category (i.e., CROW in CROWN); these stimuli yielded slower rejection times, indicating semantic activation of the embedded words. In contrast to studies on morphological processing, however, experiments on orthographically embedded words have mostly used items that are only one letter longer than the embedded word (e.g., CROW-CROWN), as a one-letter difference is how orthographic neighbors are typically defined (Coltheart et al., 1977). Consequently, they did not manipulate the morphological status of the additional letters, that is, whether they form a pseudosuffix (like -ER) or a non-morphological chunk (like -EW). Thus, the study by Nation and Cocksey (2009) points to children’s semantic activation of embedded stems, but does not explore the interaction between this phenomenon and morphological structure.

Recently, Hasenäcker, Solaja, and Crepaldi (2020b) brought together the research on the impact of morphological and orthographic information in visual word identification in Italian-speaking adults. They compared the semantic activation of embedded words in the presence and absence of a morphological structure. For this, they employed the semantic categorization paradigm of Bowers et al. (2005) but used items of the kind typically investigated in studies on morphological decomposition, that is, pseudosuffixed words like CORN-ER and nonsuffixed words like CASH-EW. They found that words were indeed harder to reject as members of a category when they embedded category-congruent word stems (i.e., rejecting CORNER as a type of food). Critically, this was the case regardless of the presence or absence of a pseudosuffix. These findings provide evidence that the lexical identification system activates the meaning of embedded word stems when the task requires semantic information, and that this is driven mostly by orthographic, not morphological, information.

The question of whether embedded word stems are only activated at an orthographic level or, rather, activation is fed forward all the way to semantics might be even more pressing for developing readers, considering the important role of morphology in reading development (for a review, see Levesque et al., 2020). In the present study, we adopted the approach of Hasenäcker et al. (2020b), and applied it to a group of 80 Italian child readers from grades 3, 4, and 5. To our knowledge, this presents the first investigation of children’s reading of pseudosuffixed words in an overt, non-priming setting, thus focusing on how these words are processed all the way up to semantics.

The peculiar feature of this paradigm is that, unlike naming, lexical decision or masked priming, children clearly need to access words’ meaning to solve the task. However, they are not explicitly asked to perform morphological operations, as is usually done in morphological awareness or complex word definition tasks. This way, morphological processing remains implicit. The goal was to find out whether *meaning* activation of embedded words in children is sensitive to morpho-orthographic structure, and whether we could detect developmental changes across grades in this respect.

As illustrated above, several studies with Italian children indicate that reading is morpheme-based, particularly for younger, less skilled readers, and when new or low-frequency words are encountered (Burani et al., 2002; Burani et al., 2008; Marcolini et al., 2011; Traficante et al., 2011). Therefore, it is possible that children, especially younger ones, will show increased sensitivity to embedded stems in pseudosuffixed items (CORNER) as compared to nonsuffixed ones (PEACE). If we find stronger congruency effects for pseudosuffixed as compared to nonsuffixed items, this would indicate that morphological structure affects embedded stem activation all the way from orthography to semantics. On the other hand, as also illustrated above, it has been argued that the presence of stems, rather than suffixes, initiates morphological segmentation in children (Traficante et al., 2011; see also Hasenäcker et al., 2017, for a similar argument). Furthermore, Nation and Cocksey (2009) found activation of embedded words without morphological structure. This may suggest that developing readers may not be differently affected by the presence or absence of pseudosuffixes, similar to the adults’ pattern uncovered by Hasenäcker et al. (2020b). If we find equally strong congruency effects for pseudosuffixed and nonsuffixed items, this would indicate that orthography alone activates the meaning of embedded stems, without a role for morphological structure. Disentangling whether or not morphological structure modulates access to embedded word meaning in children in a semantic task directly tests the assumptions of the embedded stem account, particularly on the role of morphologically structured feedback from morpho-semantics upon morphologically-blind embedded stem activation. Looking at processing in a meaning-oriented task thus promises new insights into the mechanisms behind the direct route from orthography to semantics in the developing reading system.

## Method

### Participants

Overall, 82 children participated in the present study. Twenty-five were attending third grade (11 girls, 13 boys, one no information, M_Age_ = 8.72 years, SD = 0.30), 30 were attending fourth grade (12 girls, 17 boys, one no information, M_Age_ = 9.70 years, SD = 0.39), and 27 were attending fifth grade (11 girls, 16 boys, M_Age_ = 10.89 years, SD = 0.36). Sample size was estimated based on previous studies (e.g., Nation & Cocksey, 2009). All children were Italian native speakers and had no reported diagnosis of reading- or language-related disorders as declared by the parents.

Testing took place at the International School for Advanced Studies and was part of a citizen science program, Brains@Work, which we conducted in cooperation with Medialab, the institute’s science communication partner (Zampieri, 2018). In this program, school classes visited our institute to learn about science and take part in experiments. Children were tested in threes, in quiet rooms, each of them accompanied by a trained experimenter. Signed informed consent was obtained from the parents or other legal guardians prior to the visit and oral consent was obtained from the children upon the start of the experiment. The procedure was approved by the local ethics committee.

### Material

We used the exact same material as in Hasenäcker et al. (2020b), Experiment 1. This consisted of 40 Italian nouns as carrier words, each containing an embedded word that belonged to one of six categories (animal – eight items, body part – eleven, food – eight, house – three, landscape – seven, person – three). Half of the carrier words had a pseudosuffix after the embedded word (e.g., -ONE in BURRONE), while half did not (e.g., -ACE in, RAPACE bird of prey). The embedded word was always a noun itself and was embedded at the beginning of the carrier word (e.g., BURRONE, ravine, containing BURRO, butter). Carrier words were six to ten letters long (*M* = 7.35, *SD* = 0.95), while embedded words were four to six letters long (*M* = 4.70, *SD* = 0.61). Each embedded word was of higher frequency (log-scale: *M* = 3.35, *SD* = 0.64) than its carrier word (log-scale: *M* = 2.20, *SD* = 0.62; cf. Nation & Cocksey, 2009). The length and frequency of the carrier and the embedded word were roughly equal across pseudosuffixed and nonsuffixed items (cf. Table 5).

It is worth noting that the word-final (inflectional) vowel can change or drop in Italian when a suffix is added to a stem. For example, the word TAZZA, cup, can take the diminutive suffix -INA to form the word TAZZINA (not TAZZAINA). Accordingly, some of our items preserved the final vowel of the target word in the prime (e.g., GOMITO-GOMITOLO), while this was not the case for others (e.g., POLLO-POLLICE). This applies to both the pseudosuffixed and nonsuffixed items. We controlled statistically for this variation by adding this factor as a covariate in the analyses.

Two counterbalanced experimental lists were constructed from the carrier words, such that each embedded word was assigned to its congruent category in one list and to another category in the other list. The experiment had thus a 2 × 2 design, with Congruency (category-congruent vs. category-incongruent as within-items, between-participants) and Ending (pseudosuffix vs. nonsuffix as between-items, within-participants) as crossed independent variables.

In addition to the 40 carrier words, which were the items of interest and required NO answers in the experiment, 40 additional words were selected, among the members of the six categories used in the experiment, to serve as YES–response filler trials. The fillers were matched in length (*M* = 7.43, *SD* = 1.20) and neighborhood size (*M* = 1.82, *SD* = 0.34) to the carrier words, and their frequency was between that of the carrier words and that of the embedded words (log-scale: *M* = 2.48, *SD* = 0.42). A full list of stimuli is presented in the Appendix.

### Procedure

The experiment was run using OpenSesame (Mathôt et al., 2012). The children were asked to categorize each word as quickly and accurately as possible by pressing one of two colored buttons on an Arduino response box. Each word was preceded by a fixation cross in the middle of the screen for 800 ms, followed by a blank screen for 350 ms, followed by the target word in lowercase letters. Target words were presented in the middle of the screen and remained there until button press, or for a maximum of 2,500 ms if no button press was made. The children received feedback for 500 ms after each trial (a happy or sad smiley).

Stimuli were presented in blocks by category, and the order of blocks in the experiment was randomized across participants. Each block started with the presentation of the relevant category label, which appeared in blue, uppercase letters in the middle of the screen. The experimenters read the category to the children and then initiated the trials once they were sure the children had understood the category. Two practice blocks (with the category “vehicle” and “weather”) were included at the beginning of the experiment with six trials each. The procedure was adapted from Hasenäcker et al. (2020b) with the only difference that the instructions were given orally to the children in addition to the written form.

The entire experiment took about 15 min to complete.

### Analysis

Analyses were carried out in R (R Core Team, 2014). Given that the error rate in the task was rather high (19.41% for all trials, 26.59% for trials of interest), we only excluded two participants and three items whose overall accuracy was below 50%. Further data cleaning on the level of single data points was done for the response-time analysis: incorrect responses (23.34%) were of course excluded, as were response times faster than 200 ms (0.22%). We decided to logarithmically transform the response times in order to normalize the distribution of the residuals (Baayen & Milin, 2010) based on inspection of the Box-Cox plot (*MASS package*; Venables & Ripley, 2002). Moreover, model-based outlier trimming was done by fitting a simple model with only random effects and excluding all data points with residuals exceeding 2.5 SD (1.77%; Baayen & Milin, 2010).

Error data and cleaned, log-transformed response times were analyzed using (generalized) linear mixed-effects modelling as implemented in the lme4 package (Bates et al., 2015). Models included Congruency (category-congruent embedded word vs. category-incongruent embedded word), Ending (pseudosuffix vs. nonsuffix), Grade (3, 4, 5), and their interaction as categorical fixed effects, using sum coding (i.e., comparing the mean of the dependent variable for a given level to the overall mean of the dependent variable, cf. Schad et al., 2020. Models also included Embedding (complete vs. stem) as a control variable. Finally, models included random intercepts for Subject, Item, and Category. We tested overall effects using the Type III sum of squares and χ^2^ Wald tests. Post hoc contrasts were calculated using the *emmeans* package (Lenth, 2019). Full model outputs are given in the Appendix.

## Results

**Fig. 1:**
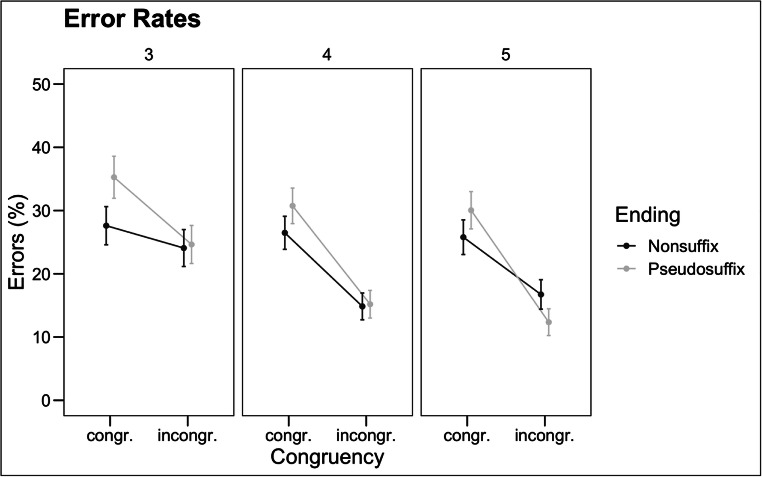
Raw error rates in the different conditions for each grade. Error bars represent standard deviations

Analysis of the error rates indicated a significant main effect of Congruency (χ^2^ = 60.26, *z* = -7.76, *p* < .001). The effect of Congruency was modulated by Grade (χ^2^ = 4.36, *p* = .034): the effect of congruency was present in all grades (grade 3: *ΔER* = 7.1%, *z* = -2.45, *p*=.014; grade 4: *ΔER* = 13.6%, *z* = -5.68, *p* < .001; grade 5: *ΔER* = 13.4%, *z* = -5.34, *p*<.001), and it became significantly stronger from grade 3 to grade 4 (*z* = 2.24, *p* = .025). Importantly, there was also an interaction between Congruency and Ending (χ^2^ = 6.30, *z = 2.51, p* = .012): the effect of congruency for pseudosuffixed words (*ΔER* = 14.6%, *z* = -6.91, *p* < .001) was stronger than that for nonsuffixed words (*ΔER* = 8.1%, *z* = -3.38, *p* < .001). The three-way interaction between Congruency, Grade, and Ending failed to reach significance (χ^2^ = 1.16, *p* = .560). The pattern of results, based on the raw means, is illustrated in Fig. 1.

**Fig. 2:**
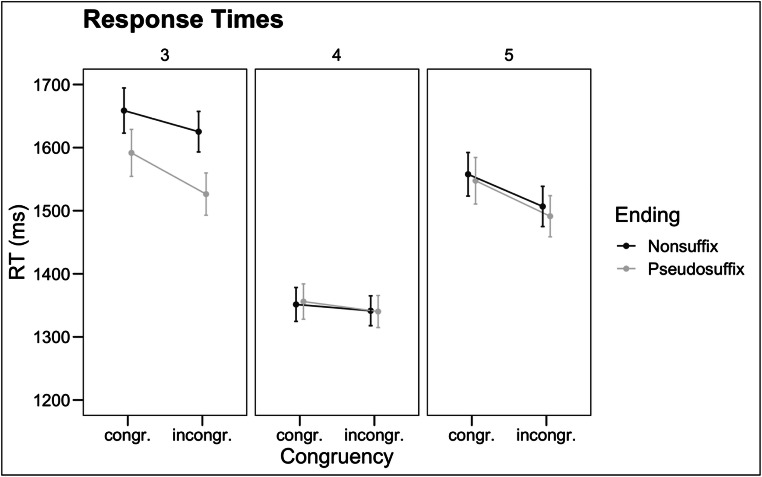
Raw response times in the different conditions for each grade. Error bars represent standard deviations

Analysis of the response times indicated a significant main effect of Congruency (χ^2^ = 9.32, *t* = 3.05, *p* = .002): rejection times were longer when the embedded word was category-congruent than when it was category-incongruent (*ΔRT* = 39 ms). There was also a main effect of Grade (χ^2^ = 11.20, *p* = .004): children in grade 4 were faster than children in grade 3 (*ΔRT* = 253 ms, *z* = 3.30, *p*<.001) and were also faster than children in grade 5 (*ΔRT* = 179 ms , *z* = 2.00, *p* = .05). No other effects reached significance (all χ^2^ < 5, all *p* > .09). The pattern of results, based on the raw means, is illustrated in Fig. 2.

We did not find a three-way interaction between Congruency, Grade, and Ending. Even more relevant to our research question, Congruency did interact with Ending in the error rate data, but not in response times. A problematic aspect of traditional frequentist null-hypothesis significance testing (NHST) is that no reliable conclusion can be drawn from non-significant results: it cannot be decided whether a null-effect counts against a theory or just indicates insensitivity of the data (e.g., low power, high standard errors). One option to address this issue is to take a Bayesian approach; the Bayes factor provides an especially easy, accessible, and sensitive option to this (Dienes, 2014, 2016). The Bayes factor is a continuous measure quantifying how likely the data are under one hypothesis compared to another. For this, two models can be directly compared, one that includes the effect of interest (H1) and one that does not (H0). The resulting ratio (BF10) can vary between 0 and infinity, with values closer to zero being in favor of H0 (usually below 1/3), values around 1 being non-conclusive (similar support of H1 and H0), and larger values (usually above 3) being in favor of H1 (for a detailed classification scheme, see Lee & Wagenmakers, 2013, based on Jeffreys, 1961). Hence, the Bayes factor allows us to overcome the asymmetry of NHST related to the fact that absence of evidence is not evidence of absence. Also, the concept of power in the NHST sense (that is, to control type II error, i.e., erroneously accepting the null hypothesis) is futile for the Bayes factor calculation, because it uses the data itself to determine the sensitivity in distinguishing the null and alternative hypothesis (Dienes, 2014). This is a very convenient property in cases like the present one, where the availability of items is limited by the language itself and by the design of the experiment.

Therefore, we resorted to Bayes factor (BF) analyses (Dienes, 2014, 2016), using the R package *BayesFactor* (Morey & Rouder, 2018). In our BF analyses, we used the full model including the three-way interaction of Congruency × Ending × Grade, and also fitted a model without this interaction. Comparing these two models, we obtain a BF close to zero, both for error rates (BF10 = 0.017 ± 11%) and for response times (BF10 = 0.019 ± 6%). Following the classification by Lee and Wagenmakers (2013), these BFs can be counted as “very strong” evidence against a model including the interaction. That is, such an effect really does not seem to be there. Turning to the two-way interaction between Congruency and Ending in the response-time analyses, the BF again provided strong evidence against the existence of such an effect: we compared a model with all two-way interactions against one with Congruency × Ending, and the BF was very close to zero (BF10 = 0.081 ± 6%). Therefore, it seems that there is indeed an interesting difference between response times, where the embedded-word cost is independent of the presence of a suffix, and error rates, where instead children are differentially affected by embedded stems according to the morphological structure of the stimulus.

## Discussion

The goal of this study was to see whether children’s ability to activate meaning from orthographically embedded words (Nation & Cocksey, 2009) is influenced by morphological structure. To this end, we adopted the same approach as in Hasenäcker et al.’s study with adults (2020b), and presented children from grades 3, 4, and 5 with words that could be either morphologically structured (pseudosuffixed, e.g., CORN-ER) or not (e.g., CASH-EW). This also presents the first investigation of children’s reading of pseudosuffixed words in an overt, non-priming setting, thus focusing on how these words’ morpho-orthographic structure influences processing all the way up to semantics.

We observed an effect of category congruency in both accuracy and response times, for children across all grades: words were harder to reject when the embedded word was congruent with the given category (e.g., CORNER with the embedded word CORN in the category “food”). This indicates that children activate the semantics of an embedded word from orthography, even when this is detrimental for the task demands. This is in line with the embedded word activation observed in Nation and Cocksey (2009) for 7-year-old, English-speaking children. It further extends these findings to the Italian language and carrier words that are more than one letter longer than the embedded stems.

There is evidence that developing readers differ cross-linguistically in how they use morphology during reading (e.g., Casalis et al., 2015; Mousikou et al*.*, 2020). Cross-linguistic differences are necessarily linked to characteristics of the languages. The characteristics that intuitively seem most important are morphological complexity and orthographic transparency (i.e., consistency of letter-sound correspondences). Surprisingly, evidence from direct cross-linguistic comparisons suggests that orthographic transparency, rather than morphological complexity, influences the extent to which morphology is used by developing readers. Casalis et al. (2015) report more benefit from morphological structure in the more transparent French compared to less transparent English orthography. According to recent evidence by Mousikou et al. (2020), however, readers in the less transparent English orthography benefitted more from morphological access than readers from the more transparent languages French, German, and Italian. In our study, we found that children access stems even in the shallow Italian orthography, similarly to what has been found earlier for the English orthography (Nation & Cocksey, 2009). This suggests that stem activation could be a core mechanism that readers draw upon regardless of the transparency of the orthographic system of their language.

Importantly, embedded words were activated not only in one-letter different neighbors, as in Nation and Cocksey’s (2009) study, but even when the carrier word was more than one letter longer than the embedded stems – actually even up to five letters longer. This shows how much activation spreads through the lexicon: even embedded words that are quite dissimilar from the carrier words in terms of total orthographic overlap are activated. A wider definition of orthographic neighborhood would surely have consequences for theories of orthographic coding in reading development. The lexical tuning hypothesis (Castles et al. 2007), for example, suggests that throughout development children refine their orthographic representations in order to better distinguish between direct substitution (e.g., CAT-HAT) and transposition (e.g., TRIAL-TRAIL) neighbors. These kinds of theories, which are built on a rather narrow neighbor definition, would have to adjust their assumptions. Theories of reading and its development that incorporate orthographic processing based on bigram coding (e.g., Grainger & Ziegler, 2011) could be better suited to accommodate our findings because they assume that orthographic activation can occur based on overlapping bigrams. However, in those theories, there should be a limit to the length difference between embedded and carrier words that still gives rise to activation, at least once the non-overlapping bigrams outnumber the overlapping ones. Our results may thus point to the need for new ways to define neighborhoods. One interesting option could be through networks in which direct connections reflect the typical one-letter neighbor, but activation spread can also be explained by additional measures, such as path length between two words, that is, the number of words that have to be crossed to get from one word (e.g., EAT) to another one (e.g., COAT) by changing one letter each time (e.g., EAT-FAT-CAT-COAT; cf. Siew, 2018). Further research in this direction is clearly warranted.

Interestingly, the congruency effect that we found can also shed light on a question raised by Dawson et al. (2018). The authors found similar response times to suffixed and nonsuffixed nonwords (e.g., EARIST vs. EARILT) in their lexical decision task with English-speaking children, and note that their study does not allow us to discern whether this means that children identify stems in *neither* types of items or in *both* types of items. The main effect of congruency in the response times in our study is clear evidence that children indeed identify the stem across both types of items.

Turning to the role of morphological structure, we found that children’s response times were not modulated by the type of ending, that is, it took equally long to reject category-congruent embedding words regardless of whether the additional letters were a pseudosuffix (e.g., -ER in CORNER) or a nonsuffix (e.g., -CE in PEACE). Because traditional frequentist statistics do not allow us to reject a hypothesis on the basis of a non-significant effect, we additionally conducted Bayes factor analyses. These yielded “strong evidence” (following the BF classification system of Lee & Wagenmakers, 2013) that the factor Ending does not influence response times on words with congruent embeddings. This indicates that children identify stems regardless of the morphological status of word endings – and this slows down semantic categorization responses, exactly like the skilled adult readers in the study by Hasenäcker at al. (2020b). It also supports the idea that stems, rather than suffixes, are the driving force of word segmentation (Traficante et al., 2011). Such a crucial role for the stem, especially in developing readers, has been suggested recently at the orthographic level based on findings from masked priming lexical decision studies (Grainger & Beyersman, 2017). Our results support this idea and additionally suggest that the orthographic stem activation is fed forward all the way up to the semantic level. This also indicates that the activation of stems in pseudosuffixed words is not a curious phenomenon triggered by a very artificial masked priming technique, but has strong relevance in reading for meaning, which is the more natural case. In fact, Amenta et al. (2015) found similar stem access in an even more natural sentence-reading paradigm in adults, regardless whether the stem contributed to the whole-word meaning or not. Hence, stems are not only activated as familiar strings of letters, but as units providing a direct orthography-to-semantics link.

Notably, we did observe an interaction with ending in children’s error rates. Children more often miscategorized a word with a category-congruent embedded stem and a pseudosuffix ending (e.g., -ER in CORNER) as compared to a word with a category-congruent embedded stem ending with a nonsuffix (e.g., -CE in PEA–CE). This suggests that the children, in contrast to the adults in Hasenäcker et al. (2020b), are influenced by the morphological surface structure of the word in making their category decision: they are more often misguided by a pseudosuffix.

Discrepancies between response times and error rates for effects of morphological structure in children’s visual word identification have been reported previously. In their lexical decision task with suffixed and nonsuffixed nonwords in English (e.g., EARIST vs. EARILT), Dawson et al. (2018) found that, while responses times were similar for both types of items, children and younger adolescents made more errors with suffixed nonwords (e.g., EARIST) than nonsuffixed nonwords (e.g., EARILT). Also, findings from Casalis et al. (2015) indicated that English-speaking children were less accurate in rejecting nonwords comprising a stem and a suffix in a lexical decision task, while no such difference was present in the response times. Similarly, Traficante et al. (2011) found that in the reading aloud of pseudowords by Italian children, the presence of a suffix affected only error rates, not response times. Dawson et al. (2018) explain the discrepancy in their findings by suggesting that children rely more heavily on explicit morphological knowledge in their judgments following the identification of a stem. Our experiment corroborates this idea: the congruency effect in the response times shows that children identify the stem across both types of items and the interaction with ending in the error rates indicates that children use morphological surface structure *after* identification of the embedded stem – and often this greater reliance on explicit morphological knowledge misguides their decision.

The idea that the children used an explicit morphological strategy in their decision-making (as reflected by the Congruency × Ending effect in the error rates) is further supported by a feature of the experimental design: the carrier words were not of high frequency and, as a consequence, several children might not have known the words very well, as the fairly high overall error rate suggests. When children were unsure about a word, it is possible that they resorted to the word’s morphological surface structure. As a consequence, they misinterpreted the pseudosuffixed words as truly suffixed words in many cases. For example, when encountering a low-frequency word like GOMITOLO, bundle, in the category body part, they might have interpreted it as the much more frequent word GOMITO, elbow, with the diminutive suffix -OLO, thus meaning something like “little elbow.” This strategy of trying to infer the meaning of unknown words (e.g., PERMEATED) by relying on the known words contained in them (e.g., MEAT) has also been explicitly reported by the participants of a study on adult second language learning of English (Nassaji, 2003) and has been demonstrated to be a strategy used by children in vocabulary acquisition (Bertram et al., 2000). Indeed, such a strategy also makes perfect sense for dealing with unfamiliar words in the elementary school years, where, in fact, morphologically complex words make up for the majority of children’s newly encountered words in reading (Anglin, 1993; Segbers & Schroeder, 2017). Similarly, Marcolini et al. (2011) found that word frequency affected the probability of morpheme-based reading: they observed an advantage especially of low-frequency morphologically complex words in Italian children’s reading aloud.

The pattern of results that we found, with no role for (pseudo)morphological structure in the response times but a modulation from (pseudo)morphological structure in the error rates, can possibly be explained within the embedded stem activation framework (Grainger & Beyersman, 2017). This account assumes early morphologically blind activation of embedded words, which can be modulated by feedback from morpho-semantic representations, which are sensitive to (pseudo)morphological structure and the principle of full decomposition. It seems that the morpho-semantic feedback in children manifests particularly in the error rates. Considering that the feedback mechanism is thought to be especially important for developing readers in order to help them understand unfamiliar words containing familiar stems, its emergence in the explicit decisions itself, rather than in its duration, might be expected.

It is also noteworthy that the congruency effect in the error rates increased from grade 3 to grade 4, but the influence of the ending did not change over grades, as the Bayes factor analyses additionally confirmed. This again shows parallels to the study by Dawson et al. (2018), where the effect of the type of ending (suffixed vs. nonsuffixed) did not differ in magnitude between children and younger adolescents, but only differed between younger adolescents and older adolescents. This suggests that developmental changes of the influence of ending occur at a later point in reading development than we captured in the present study. One possible drawback of our study is that power in the separate grades is rather on the low side (although comparable to previous studies). Consequently, we suggest a cautious interpretation of changes between grades, and that we should focus more on the effects that we see for the larger entire sample. However, the results of the additional BF analyses are further reassurance that the null effects we observed are likely not due to low power. An interesting endeavor for future studies would be to cover the age range between elementary school and adulthood to test at what point in development morphological surface structure stops to have an influence on the error rates.

The present study investigated the use of orthographic and morphological information in word meaning activation in children and yielded two important findings. Elementary school children already activate semantics of embedded words based on orthography, not morphological structure per se: similar to adults (Hasenäcker et al., 2020b), they “see” the embedded word regardless of the nature of the word ending, and this slows down semantic categorization responses. This expands the results of Nation and Cocksey (2009), and importantly qualifies the workings of the orthography-morphology interface in developing readers. However, the morphological surface structure is not simply discarded: its effect emerges strategically at the decision-level, manifesting as an effect in the error rates, especially for words whose meaning may not be familiar to the children, and therefore is sought in the word’s (pseudo)morphological structure. This highlights an interesting dichotomy in the role of morphological structure between automatic/implicit activation (reflected in the response times) and explicit strategies (reflected in the error rates).

## Appendix

**Table 1:** Example stimuli in all conditions in English and Italian. Embedded words are underlined, categories are given in uppercase

	NO-answers				YES-answers
	Category-congruent		Category-incongruent		
	Pseudosuffix Ending	Nonsuffix Ending	Pseudosuffix Ending	Nonsuffix Ending	
Stimulus	*burrone*, ravine	*rapace*, bird of pray	*burrone*, ravine	*rapace*, bird of pray	*arancia*, orange
Embedded word	*burro*, butter	*rapa*, turnip	*burro*, butter	*rapa*, turnip	
Category	Food	Food	Body part	Body part	Food
Comparable example in English	corner	peace	corner	peace	carrot

**Table 2:** Model output from analysis of accuracy data. χ^2^-, z-, and p-values for fixed effects were calculated using Type 3 Wald Chi-square tests. Post-hoc contrasts were calculated using multiple pairwise comparisons as implemented in 95% confidence intervals were calculated using the Wald method

Fixed effects						
	χ^2^	Est/Beta	SE	95% CI	z	p
Intercept	49.52	1.40	0.20	1.01 – 1.79	7.03	<0.001
Embedding	< 1	-0.003	0.10	-0.20 – 0.20	-0.03	0.974
Congruency	60.26	-0.38	0.05	-0.47 – -0.28	-7.76	<0.001
Ending	< 1	0.04	0.10	-0.15 – 0.24	0.43	0.664
Grade	4.36					0.113
Congruency X Ending	6.32	0.14	0.05	0.03 – 0.24	2.51	0.012
Congruency Pseudosuffix		1.03	0.15	0.74 – 1.32	6.91	<0.001
Congruency Nonsuffixed		0.48	0.14	0.20 – 0.76	3.38	<0.001
Congruency X Grade	6.76					0.034
Congruency Grade 3		0.40	0.16	0.08 – 0.73	2.45	0.014
Congruency Grade 4		0.92	0.16	0.60 – 1.23	5.68	<0.001
Congruency Grade 5		0.95	0.18	0.60 – 1.29	5.34	<0.001
Ending X Grade	1.67					0.433
Ending Grade 3		0.22	0.24	-0.25 – 0.69	0.91	0.361
Ending Grade 4		0.13	0.24	-0.34 – 0.59	0.54	0.591
Ending Grade 5		-0.08	0.25	-0.57 – 0.40	-0.34	0.731
Congruency X Ending X Grade	1.16					0.560
Congruency Pseudosuffix Grade 3		0.64	0.24	0.18 – 1.11	2.70	0.007
Congruency Nonsuffix Grade 3		0.16	0.24	-0.30 – 0.63	0.69	0.491
Congruency Pseudosuffix Grade 4		1.08	0.23	0.62 – 1.54	4.60	<0.001
Congruency Nonsuffix Grade 4		0.75	0.23	0.30 – 1.21	3.26	0.001
Congruency Pseudosuffix Grade 5		1.36	0.27	0.84 – 1.88	5.11	<0.001
Congruency Nonsuffix Grade 5		0.53	0.24	0.06 – 1.01	2.19	0.029
Random Effects						
			Variance		S.D.	
Participant (Intercept)			0.68		0.82	
Word (Intercept)			0.23		0.48	
Block (Intercept)			0.13		0.36	
Model fit						
R^2^			Marginal		Conditional	
			0.05		0.28	

Key: p-values for fixed effects calculated using Type III Wald Chi-square tests

Pairwise comparisons computed using emmeans()

Confidence Intervals have been calculated using the Wald method

Model equation: Accuracy ~ Congruency * Ending * Grade + (1 | Participant) + (1 | Word) + (1 | Block)

**Table 3:** Model output from analysis of response time data (log-transformed). |^2^-, z-, and p-values for fixed effects were calculated using Type 3 Wald Chi-square tests

Fixed effects						
	χ^2^	Est/Beta	SE	95% CI	t	p
Intercept	65917	7.275	0.028	7.220 – 7.331	256.75	<0.001
Embedding	< 1	-0.006	0.013	-0.033 – 0.020	-0.48	0.630
Congruency	9.32	0.013	0.004	0.005 – 0.021	3.05	0.002
Ending	< 1	0.005	0.013	-0.021 – 0.031	0.37	0.566
Grade	11.20					0.004
Congruency X Ending	2.20	-0.007	0.005	-0.016 – 0.002	-1.48	0.138
Congruency Pseudosuffix		-0.039	0.012	-0.064 - -0.014	-3.04	0.002
Congruency Nonsuffixed		-0.012	0.013	-0.036 – 0.013	-0.96	0.336
Congruency X Grade	< 1					0.677
Congruency Grade 3		-0.033	0.016	-0.064 - -0.002	-2.08	0.038
Congruency Grade 4		-0.016	0.013	-0.042 – 0.010	-1.18	0.239
Congruency Grade 5		-0.028	0.014	-0.055 - -0.001	-2.02	0.043
Ending X Grade	4.75					0.093
Ending Grade 3		0.030	0.030	-0.030 – 0.090	1.01	0.317
Ending Grade 4		-0.014	0.029	-0.071 – 0.044	-0.48	0.637
Ending Grade 5		0.013	0.029	-0.045 – 0.071	0.44	0.661
Congruency X Ending X Grade	< 1					0.623
Congruency Pseudosuffix Grade 3		-0.057	0.023	-0.102 - -0.011	-2.44	0.015
Congruency Nonsuffix Grade 3		-0.009	0.022	-0.052 – 0.035	-0.40	0.690
Congruency Pseudosuffix Grade 4		-0.029	0.020	-0.068 – 0.010	-1.46	0.144
Congruency Nonsuffix Grade 4		-0.002	0.019	-0.040 – 0.035	-0.13	0.899
Congruency Pseudosuffix Grade 5		-0.032	0.020	-0.072 – 0.008	-1.55	0.120
Congruency Nonsuffix Grade 5		-0.025	0.020	-0.064 - -0.014	-1.24	0.216
Random Effects						
			Variance		S.D.	
Participant (Intercept)			0.05		0.22	
Word (Intercept)			0.005		0.07	
Block (Intercept)			0.0003		0.18	
Model fit						
R^2^			Marginal		Conditional	
			0.07		0.62	

Post hoc contrasts were calculated using multiple pairwise comparisons as implemented in . 95% confidence intervals were calculated using the Wald method

Key: p-values for fixed effects calculated using Type III Wald Chi-square tests

Pairwise comparisons computed using

Confidence Intervals have been calculated using the Wald method

Model equation: log(ResponseTime) ~ Congruency * Ending * Grade + (1 | Participant) + (1 | Word) + (1 | Block)

**Table 4:** Full list of stimuli items used in the experiment

Target word	Embedded word	Ending	Congruent category	Incongruent Category	Answer
canestro	cane	nonsuffix	ANIMAL	LANDSCAPE	NO
pollice	pollo	nonsuffix	ANIMAL	LANDSCAPE	NO
falcata	falco	pseudosuffix	ANIMAL	LANDSCAPE	NO
focaccia	foca	pseudosuffix	ANIMAL	LANDSCAPE	NO
merletto	merlo	pseudosuffix	ANIMAL	LANDSCAPE	NO
mulino	mulo	pseudosuffix	ANIMAL	LANDSCAPE	NO
polpaccio	polpo	pseudosuffix	ANIMAL	LANDSCAPE	NO
tassello	tasso	pseudosuffix	ANIMAL	LANDSCAPE	NO
colloquio	collo	nonsuffix	BODY PART	FOOD	NO
dentice	dente	nonsuffix	BODY PART	FOOD	NO
gambero	gamba	nonsuffix	BODY PART	FOOD	NO
maniaco	mano	nonsuffix	BODY PART	FOOD	NO
manovra	mano	nonsuffix	BODY PART	FOOD	NO
ossido	osso	nonsuffix	BODY PART	FOOD	NO
pellicola	pelle	nonsuffix	BODY PART	FOOD	NO
vischio	viso	nonsuffix	BODY PART	FOOD	NO
gomitolo	gomito	pseudosuffix	BODY PART	FOOD	NO
mentore	mento	pseudosuffix	BODY PART	FOOD	NO
visone	viso	pseudosuffix	BODY PART	FOOD	NO
carnevale	carne	nonsuffix	FOOD	BODY PART	NO
rapace	rapa	nonsuffix	FOOD	BODY PART	NO
riserbo	riso	nonsuffix	FOOD	BODY PART	NO
salamandra	salame	nonsuffix	FOOD	BODY PART	NO
tortura	torta	nonsuffix	FOOD	BODY PART	NO
burrone	burro	pseudosuffix	FOOD	BODY PART	NO
lattina	latte	pseudosuffix	FOOD	BODY PART	NO
panico	pane	pseudosuffix	FOOD	BODY PART	NO
scalogno	scala	nonsuffix	HOUSE	PERSON	NO
camerata	camera	pseudosuffix	HOUSE	PERSON	NO
salario	sala	pseudosuffix	HOUSE	PERSON	NO
costola	costa	nonsuffix	LANDSCAPE	ANIMAL	NO
marchio	mare	nonsuffix	LANDSCAPE	ANIMAL	NO
passero	passo	nonsuffix	LANDSCAPE	ANIMAL	NO
costanza	costa	pseudosuffix	LANDSCAPE	ANIMAL	NO
montone	monte	pseudosuffix	LANDSCAPE	ANIMAL	NO
rivale	riva	pseudosuffix	LANDSCAPE	ANIMAL	NO
valletto	valle	pseudosuffix	LANDSCAPE	ANIMAL	NO
magagna	mago	nonsuffix	PERSON	HOUSE	NO
mattone	matto	pseudosuffix	PERSON	HOUSE	NO
pretesa	prete	pseudosuffix	PERSON	HOUSE	NO
anatra			ANIMAL		YES
capriolo			ANIMAL		YES
coccinella			ANIMAL		YES
farfalla			ANIMAL		YES
leopardo			ANIMAL		YES
lucertola			ANIMAL		YES
pavone			ANIMAL		YES
scoiattolo			ANIMAL		YES
caviglia			BODY PART		YES
guancia			BODY PART		YES
labbro			BODY PART		YES
muscolo			BODY PART		YES
ombelico			BODY PART		YES
tallone			BODY PART		YES
addome			BODY PART		YES
ascella			BODY PART		YES
ciglio			BODY PART		YES
torace			BODY PART		YES
unghia			BODY PART		YES
arancia			FOOD		YES
banana			FOOD		YES
biscotto			FOOD		YES
caramella			FOOD		YES
carciofo			FOOD		YES
lasagna			FOOD		YES
mandarino			FOOD		YES
minestra			FOOD		YES
attico			HOUSE		YES
balcone			HOUSE		YES
solaio			HOUSE		YES
cascata			LANDSCAPE		YES
palude			LANDSCAPE		YES
pascolo			LANDSCAPE		YES
pianura			LANDSCAPE		YES
ruscello			LANDSCAPE		YES
scogliera			LANDSCAPE		YES
vulcano			LANDSCAPE		YES
macellaio			PERSON		YES
meccanico			PERSON		YES
muratore			PERSON		YES

**Table 5:** Mean length and frequency of embedded words and carrier words in the pseudosuffixed and nonsuffixed condition (standard deviation in parentheses)

	Pseudosuffixed items	Nonsuffixed items
Embedded Word Length (number of letters)	4.80 (0.62)	4.60 (0.60)
Embedded Word Frequency (log10)	3.10 (0.63)	3.60 (0.55)
Carrier Word Length (number of letters)	7.25 (0.85)	7.45 (1.05)
Carrier Word Frequency (log10)	2.18 (0.51)	2.23 (0.73)
